# The Reduction Reaction Behavior of Steelmaking Dusts with Lignin under Different Atmospheres

**DOI:** 10.3390/ma17133106

**Published:** 2024-06-25

**Authors:** Danuka Maduranga Wawita Widanalage Don, Timo Fabritius, Mamdouh Omran

**Affiliations:** Process Metallurgy Research Unit, Faculty of Technology, University of Oulu, Pentti Kaiteran Katu 1, 90570 Oulu, Finland; danuka.wawitawidanalagedon@oulu.fi

**Keywords:** electric arc furnace, lignin, CRC, EAF dust

## Abstract

This study investigated lignin as a reducing agent instead of fossil carbon for the reduction of zinc oxide and zinc ferrite contained in steelmaking dusts. Three types of dusts from different steelmaking processes were considered: ferrochrome converter (CRC), electric arc furnace stainless steel (EAFSS) and electric arc furnace carbon steel (EAFCS). Zinc is primarily found in zincite phases within CRC dust, while EAFSS and EAFCS dusts contain franklinite and zincite phases as Zn-bearing minerals. The proximate analysis of lignin showed that the fixed carbon content is 28.9%. Thermogravimetric (TG) analysis coupled with differential scanning calorimetry (DSC) and mass spectrometry (MS) was used to study the reduction behavior of different mixtures of lignin and steel dusts under inert and air atmospheres. Simultaneously, the minimum ratio of lignin out of three different proportions required to achieve a complete reduction of franklinite and zincite phases into metallic zinc was identified. The results indicated that a 1.1 stoichiometric amount of lignin is sufficient for the complete reduction of zinc-bearing minerals into metallic zinc. In conclusion, lignin can be used efficiently for processing steelmaking dusts.

## 1. Introduction

Steel plays a vital role in the modern world. Apart from being used as a building material, steel is the initiating material of most manufacturing activities. The researchers assume that if the metal extraction is sustainable, it will be sufficient to fulfill the requirements of a world population of 9 billion for 50 years [1]. Equally crucial is our responsibility to preserve these nonrenewable natural resources for future generations. Therefore, the efficient consumption of these metals is paramount today, as it is at any point in time. Within the steel industry, various types of steelmaking dust are generated, including electric arc furnace dust and ferrochrome dust [2]. Roughly 15–25 kg of EAF dust is generated for every ton of EAF steel produced [3]. Simply disposing of these dusts would not only exacerbate the scarcity of these valuable metals but also pose environmental toxicity concerns, especially harmful agents, including approximately 21–33% iron and 17–35% zinc [4].

The primary methods for recycling EAF dust include hydrometallurgical and pyrometallurgical techniques. Additionally, a combination of both methods and microwave reduction technology have emerged as potential approaches in recent times. The main pyrometallurgical technique for zinc reduction is the Waelz process, which is based on a carbothermic reduction of zinc oxide and the volatilization of metallic zinc [2]. However, it has several drawbacks, such as wasting iron units, consuming significant energy, and incurring high tipping and shipping costs. In the pyrometallurgical recycling of EAF dust, the fume (zinc oxide fume) generated is usually polluted by chlorine and fluorine [5,6], while the residue/slag produced often has a high iron content [7]. The most critical challenges in pyrometallurgical techniques are the complex occurrence of various metal-bearing phases in the dust, the re-oxidation of non-ferrous metals after reduction and the enrichment of hazardous impurities in the generated fume and residue [8,9]. The main limitation of hydrometallurgical process is the insolubility of franklinite phase in EAF dust. Unfortunately, the majority of Zn and Fe ions exist in the franklinite phase, which will lead to an increase the inaccuracy of this process [10,11,12].

In the recently published work, the characterization of EAF dust and improving zinc recovery from steelmaking dust by switching from conventional heating to microwave heating have been studied [2,13]. The authors have concluded that all three types of dusts contain a significant amount of zinc, primarily in the zincite and franklinite phases. Zinc content may vary from 10% to 35% within the sample according to the dust type and the process technology [13]. Moreover, graphite or anthracite, being rich in fixed carbon, shows promise as a reducing agent due to its high and pure fixed carbon content (95–99%). Previous studies have effectively utilized graphite in the reduction process of zinc using both microwave and conventional heating methods [2]. Furthermore, an extensive review of the opportunities to use biomass-based fuels in iron and steelmaking processes has been conducted [14]. The authors revealed that the fixed carbon in the biomass would have been effectively utilized for the reduction process of zinc oxide and zinc ferrite at an elevated temperature. The present study examines the suitability of lignin as a reducing agent for zinc oxide reduction, targeting zincite and franklinite phases present in steelmaking dusts. Utilizing biomass over fossil reducing agents offers key advantages, such as reducing CO_2_ emissions and fostering sustainable development. This aligns with the EU’s carbon emission reduction plan, enhancing efforts towards a greener future.

Lignin is a complex, oxygen-containing, highly branched bio polymer that forms the chief constituent of wood [15]. This is the second-most-abundant natural material on the Earth [16]. Generally, this is produced as a byproduct in the paper and bioethanol industries. It is estimated that about 1.5–1.8 billion tons of lignin are produced annually from industrial sources, and among this, 50–70 million tons are produced at pulp and paper facilities worldwide [17]. Lignocellulosic biomass consists of three major components called lignin, cellulose, and hemicellulose. Several methods, including lignoboost, enzyme hydrolysis, and yeast fermentation processes, are employed to extract lignin from biomass. Once separated from biomass, lignin can serve as an eco-friendly substitute for fossil fuels in the steel industry, acting as a reducing agent [16].

Utilizing lignin as a reducing agent in the steelmaking dust recycling process offers several advantages. Lignin’s significant fixed carbon content makes it an excellent candidate for use as a reducing agent. The emissions of CO_2_ from this process are minimal in terms of the impact on the carbon cycle, as biomass sources such as lignin have short life cycles. Contrastingly, fossil carbon sources are costly and limited, leading to increased material costs for recycling processes. Therefore, the utilization of lignin presents an environmentally friendly and sustainable alternative to fossil carbon. But it also has several difficulties. Even though lignin is readily available, especially in Europe, it has been a challenge to use it directly in the steel industry due to the high sulfur content inside the lignin. The most abundant type of lignin is sulfonate lignin, characterized by a sulfur content ranging from 3.5% to 8% (wt%). Kraft lignin is the second-most-abundant variety, containing approximately 1% to 3% (wt%) sulfur. Hydrolysis lignin, although possessing a lower sulfur content of 0% to 1% (wt%), is comparatively less available in quantity [18,19]. In this study, hydrolysis lignin is used as the reducing agent for the Zn reduction process of steel dusts. Finally, we aim to investigate the feasibility of utilizing lignin as a reducing agent for steelmaking dust. Additionally, we aim to investigate the distinctive properties of these materials and analyze the atmospheric impact on the reduction process.

## 2. Materials and Methods

In this study, three types of steel dust materials were considered: ferrochrome converter (CRC), electric arc furnace stainless steel (EAFSS), and electric arc furnace carbon steel (EAFCS) dust. CRC and EAFSS dusts were obtained from Outokumpu Tornio stainless steel plant, Finland, and EAFCS samples were obtained from Ovako Imatra, Finland. As a side stream, steelmaking dusts are generated in converters and electric arc furnaces (EAFs) and accumulated inside the baghouses [13,20,21].

The dust can be generated in different stages during the EAF process, such as the volatilization of molten steel, the projection of droplets occurs in the steel bath, bursting the droplets, and the direct flying of solid particles during the charging stage [22,23,24]. In galvanized steel processing, zinc content in the dust can reach 30% or more, and additionally, chlorides, fluorides, sulfates, and sulfides can also be observed [25]. The expected reduction reactions of zincite and franklinite with carbon are as follows:

ZnO(s) + C(s) = Zn(s) + CO(g)(1)

ZnFe_2_O_4_(s) + 4C(s) = Zn(s) + 2Fe(s) + 4CO(g) (2)

**Figure 1:**
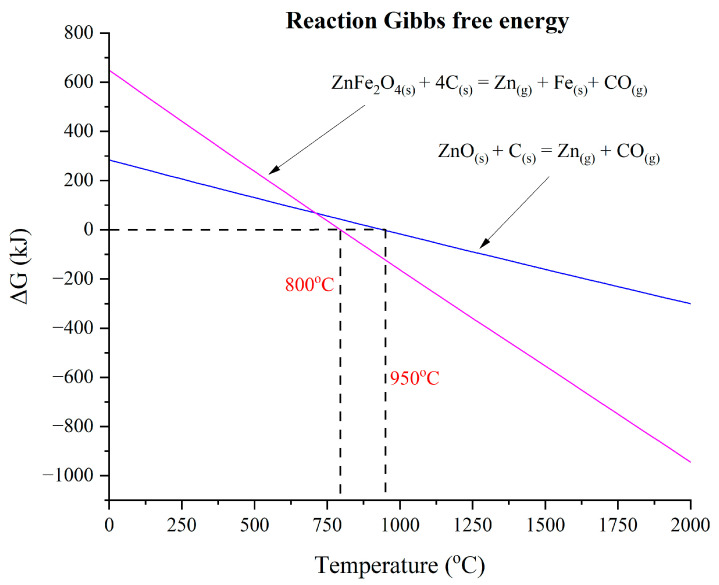
XRD results of raw dust samples of CRC, EAFSS and EAFCS modified from [2].

The Gibbs free energy calculation was considered during the experiment [2], as shown in Figure 1. These calculations were performed using FactSage (version 7.2) along with its FactPS, FToxid, and FSstel databases. Calculations were conducted for 100 g of dust at a total pressure of 1 atm. It was determined that zincite and franklinite phases spontaneously reduce to zinc at 950 °C and 800 °C.

The stoichiometric amounts were calculated using the following mass equations.

(3) $${\;m}_{c}=\frac{n_{c}}{n_{\mathrm{Zno}}}\times \frac{M_{c}}{M_{\mathrm{Zno}}}$$

(4) $$m_{C}=\frac{n_{C}}{n_{{\mathrm{ZnFe}}_{2}O_{4}}}\times \frac{M_{C}}{M_{{\mathrm{ZnFe}}_{2}O_{4}}}$$

The steel dusts were mixed with lignin and graphite according to the stoichiometric proportion required to reduce all zinc oxide and zinc ferrite contained in the dust to metallic Zn, and the mixing quantities are shown in Table 1. Graphite was used as a reference reducing agent.

The mineralogical composition was identified using a Rigaku SmartLab 9 kw X-ray diffractometer (Malvern Panalytical, Almelo, The Netherlands). The scanning range was 5–130 degrees and utilized a Co rotating anode. Phases were discerned in the diffraction pattern using Rigaku integrated X-ray powder diffraction software PDXL 2.6, relying on the ICDD PDF-4 database. A calibrated PANalytical Axios max 4 kW XRF machine (Malvern Panalytical, Almelo, The Netherlands) with a rhodium anode was used for the determination of the chemical composition of dust samples in a vacuum medium. The instrument had an automatic sample changer system for the effective processing of large amounts of samples. A LECO test was conducted to identify the carbon content of dust samples with the Leco CS230 carbon sulfur analyzer (LECO, St. Joseph, MI, USA). The morphological and microanalyses of residue of different mixtures were analyzed using a Zeiss ULTRA Plus field-emission scanning electron microscope (FE-SEM) (Carl Zeiss, Oberkochen, Germany) equipped with an energy-dispersive X-ray spectroscopy (EDX) unit for chemical analysis. A thin layer of carbon was applied using a JEE-420 Vacuum Evaporator (JEOL Ltd., Tokyo, Japan) and analyzed by using abovementioned SEM-EDS analyzer (Carl Zeiss, Germany). The thermal analysis (TG, DSC) was conducted using a NETZSCH STA 449 F3 graphite furnace (NETZSCH, Selb, Germany), which facilitates conducting TG and DSC experiments simultaneously. The volatile gas line of this machine was connected to the NETZSCH QMS 403 D mass spectrometry (MS) analyzer (NETZSCH, Selb, Germany), which provided information about the volatile materials during the TG analysis.

## 3. Results

### 3.1. Material Characterization of Raw Dust

**Figure 2:**
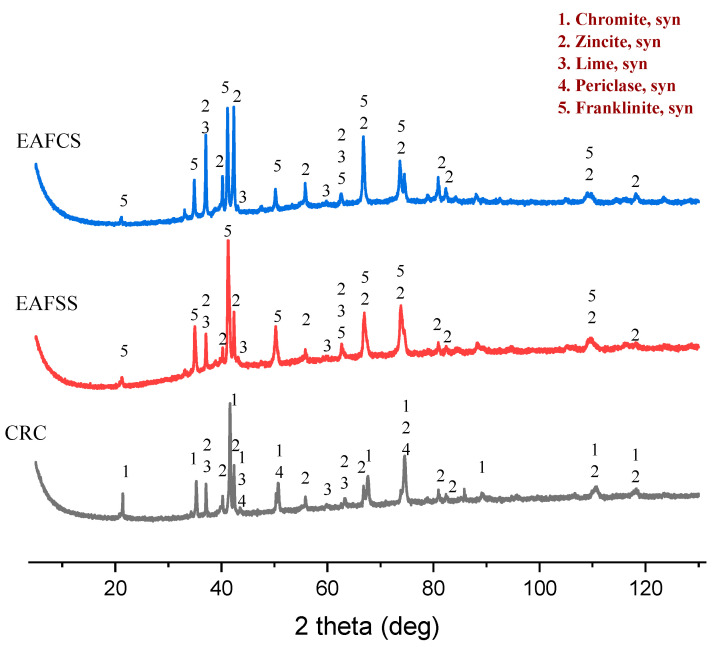
XRD results of raw dust samples of CRC, EAFSS and EAFCS.

The mineralogical composition of raw steel dust is as shown in Figure 2. All three dust samples primarily contain zincite (ZnO) as the main phase. In addition to zincite, CRC dust contains chromite (FeCr_2_O_4_), while EAFSS and EAFCS contain the franklinite phase (ZnFe_2_O_4_). Lime (CaO) is present in all three samples as a secondary phase, while periclase (MgO) is specifically found in the CRC dust.

The XRF results of steel dusts, as shown in Table 2, highlight the identification of the chemical composition of steelmaking dust samples. There are significant differences in zinc content between EAFCS and EAFSS/CRC dust, attributed to production methods and additive usage [26,27].

The ultimate analysis provides the elemental composition of the lignin, as summarized in Table 3. The composition of C, O, and H elements are 61%, 31.9%, and 6.1%, respectively, since lignin comes from organic biomass. As this undergoes no pyrolysis process, both volatile matters and moisture are present. There is significant sulfur and nitrogen content within the lignin material, which may be a more considerable factor since this adversely affects the steel-dust recycling and reusing process. The proximate analysis shows the major components of the lignin sample, including volatile matters, ash content and fixed carbon content. Table 4 shows the composition of ash content, comprising elements like Cl, F, S, among others. Understanding the ash content is crucial as these elements persist as residues within steel samples following exposure to heat [28,29,30].

**Figure 3:**
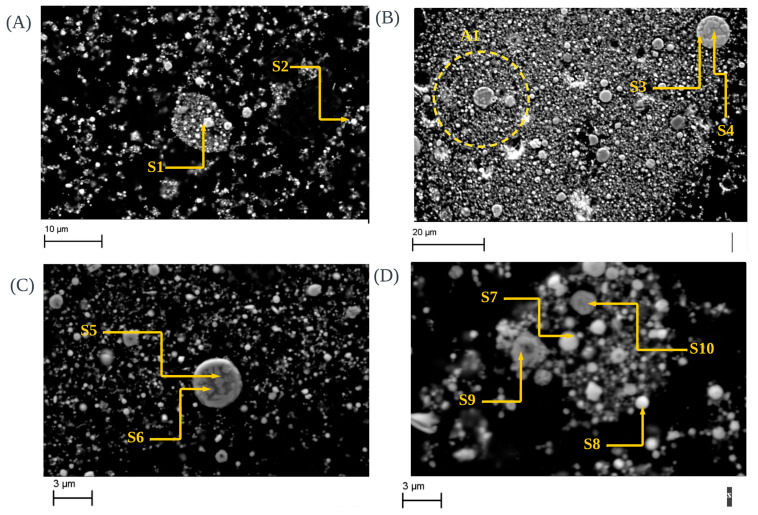
SEM images of raw CRC dust (**A**), raw EAFSS dust (**B**) and raw EAFCS dust (**C**,**D**): (i) Chromite phase covered with different fine-grained materials (Mg, Mn) (S1), presence of zincite phase (S2), (ii) Encapsulation phenomena of EAFSS dust which franklinite particles covered with fine grained particles (A1), Agglomeration of franklinite phase enclosed by glass sphere (S3 and S4) inside the EAFSS dust, (iii) Agglomeration of franklinite phase in EAFCS dust enclosed by glass sphere (S5 and S6), Presence of zincite phase (S7 and S8) and presence of franklinite phase (S9 and S10) with encapsulation phenomena up to some extent inside the EAFCS dust.

Figure 3 shows the morphological findings of raw steel dusts. As shown in Figure 3A, morphological findings indicate the presence of the chromite phase in raw CRC dust (S1). Additionally, according to Table 5, the EDX analysis further indicates high concentrations of Cr-F-O due to the presence of these irregularly shaped chromite phases. Additionally, the spectrum values suggest significant levels of zinc (Zn) and oxygen (O), along with other elements such as carbon (C), chromium (Cr), and iron (Fe), providing evidence for the existence of zincite (S2).

SEM images of raw EAFSS samples (as shown in Figure 3B) illustrate the agglomeration of franklinite particles enclosed within glass spheres (S3 and S4), confirmed by the elemental distribution, as shown in Table 5. The area covered with A1 is a clear identification of encapsulation phenomena [2]. Furthermore, SEM images of raw EAFCS dust (as shown in Figure 3C,D) exhibit the presence of zincite (S7 and S8) and franklinite phases (S5, S6, S9 and S10), with encapsulation phenomena observed to some extent. This insight into the encapsulation of franklinite phases within calcium iron-silicate glass spheres underscores the higher zinc content within the franklinite particles compared to the surrounding glass matrix, as observed by various authors [11,47,48].

### 3.2. Thermal Analysis of Raw Dust

**Figure 4:**
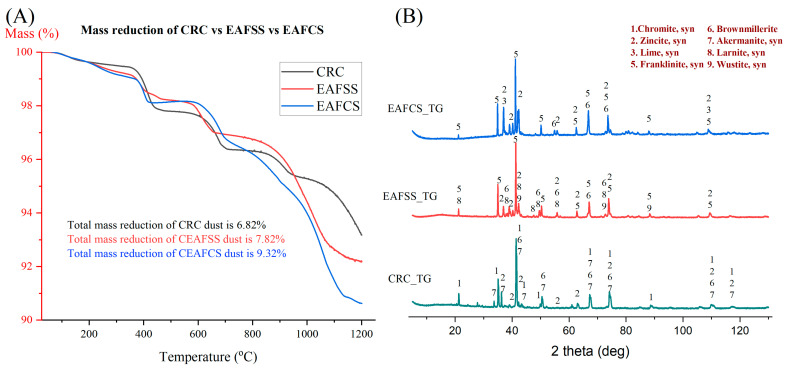
Comparison of TG—MS analysis of raw CRC, EAFSS and EAFCS dust samples and XRD analyses after TG: (**A**) comparison of TG analysis, and (**B**) XRD analysis after TG.

The thermal behavior of all three steel dust samples in an inert atmosphere (Ar) was analyzed using thermogravimetric (TG) and mass spectrometry (MS) analysis, as shown in Figure 4A. All the three raw dust samples contain four distinct stages when subjected to heating (Appendix B—Figure A3A,D,G): the evaporation process, the dihydroxylation process, the decomposition of carbonates, and the reduction stages [49]. The highest mass reduction was achieved by the EAFCS dust. This implies the highest proportion of volatile matters such as moisture, hydroxides, and carbonates inside the dust and the highest carbon content according to the XRF results in Table 2. It is possible to reduce some of the metal oxides by using this in situ carbon inside the samples. The ionic current curves belonging to m18, m28, and m44 correspond to H_2_O, CO, and CO_2_, respectively.

As shown in Figure 4B, the XRD results of raw sample residues after TG analysis show slightly similar phases of their raw samples. Both CRC raw and CRC after TG results show the presence of chromite and zincite phases. The periclase and lime phases disappear from the CRC samples when subjected to heat and recreate different phases named akermanite and brownmillerite under elevated temperatures. The EAFSS sample shows the same franklinite and zincite phases inside the residue after TG analysis, with the presence of brownmillerite, wustite and larnite phases. Wustite might appear due to the reduction process of iron oxides, if we observe the internal carbon presence inside the dust sample. The EAFCS dust also shows the presence of zincite and franklinite phases in their residues after TG analysis. Considering these factors, it was established that mere heat is insufficient for the reduction process of steelmaking dust. Therefore, it is essential to utilize a reducing agent containing an appropriate proportion of fixed carbon within the sample. Throughout this study, lignin served as the target reducing agent and was compared with a reference graphite sample to assess its efficiency as a reducing agent.

**Figure 5:**
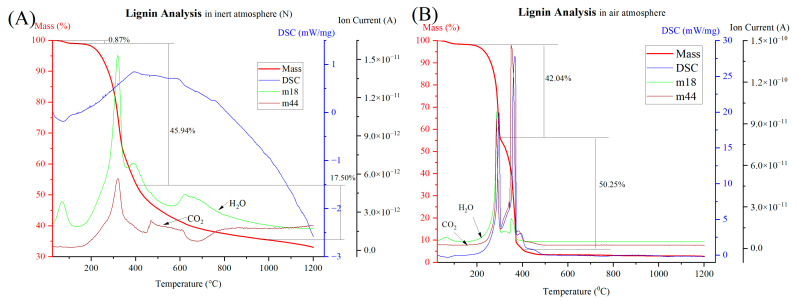
TG-DSC-MS curves of lignin sample under inert (**A**) and air (**B**) atmospheres.

TG, DSC, and MS analysis of raw lignin under air and inert atmospheres are shown in Figure 5. Under the inert atmosphere shown in Figure 5A, the TG, DSC and MS curves of the lignin sample have significantly different behavior compared to the air atmosphere. The slight mass reduction between 0 and 105 °C belongs to vaporization. The mass reduction percentage was 0.87, which was a comparatively minor loss. Notably, there was a significant fluctuation in the mass spectra of m/z18 (H_2_O) within this temperature range, suggesting the release of water vapors from the sample. The temperature between 200 and 400 °C shows the highest reaction kinetics with a 45.94% mass reduction.

During this temperature range, mass spectra curves belonging to H_2_O and CO_2_ appeared to have high fluctuation. The identified reaction phenomena for these reductions were the thermal decomposition of carbohydrates, leading to the breakdown of complex structures into simpler compounds [31,50]. This process involved the release of water vapor, carbon dioxide, and other volatile byproducts such as methane, which perfectly match to the TG, DSC, and MS data. The total mass reduction was 66.93% following heating to 1200 °C, where around 33% of mass remains as biochar.

As shown in Figure 5B, in an air atmosphere, the TG curve contained two major mass reductions occurring within the temperature ranges of 150 to 300 °C and 300 to 400 °C. The significant fluctuations of many mass curves have occurred within this temperature range, notably marked by pronounced variations in both the m18 (water vapor) and m44 (CO_2_) curves. Within the same temperature range, the DSC curve indicates two extremely elevated exothermic peaks, suggestive of the combustion process of lignin with the presence of adequate oxygen in the medium. The residue was almost zero, indicating the absence of biochar formation during the combustion process within the medium.

### 3.3. Effect of Mixing Proportion of Lignin as a Reducing Agent

The impact of different lignin mixing proportions on the zinc reduction process was examined utilizing different mixing ratios, as shown in Table 1. Additionally, the minimum lignin mixing percentage out of the three different ratios was determined for the complete reduction process of zinc-bearing oxides into metallic zinc. The study investigated the effect of lignin ratio on the reduction efficiency of zinc oxide and zinc ferrite. Three types of steel dust samples (CRC, EAFSS, and EAFCS) with three distinct mixing proportions (1.1, 1.3 and 1.5) of lignin material were analyzed, and the mass losses with respect to different temperature ranges are summarized in Table 6. The initial mass loss due to vaporization has been excluded here.

**Figure A1:**
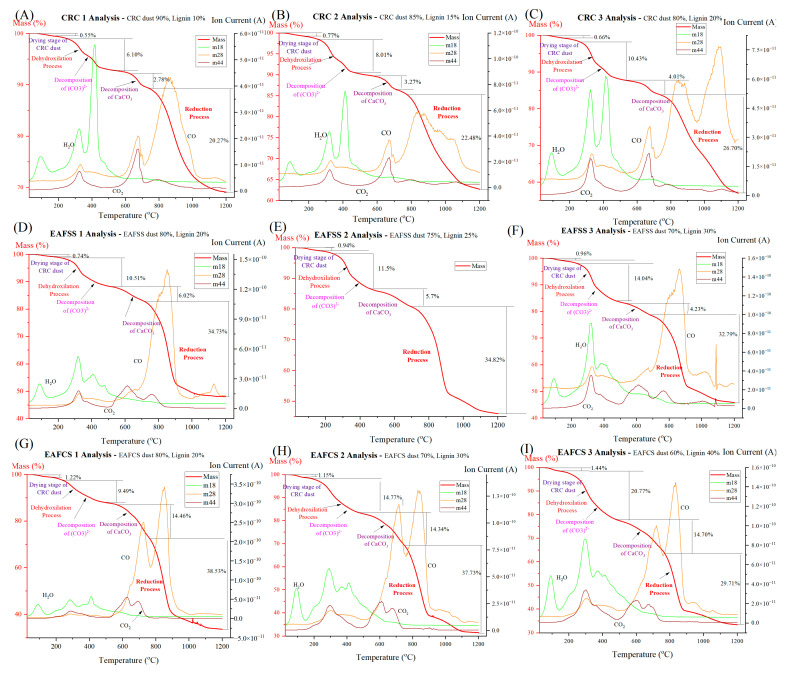
TG-MS analysis of different lignin mixtures with different dust samples under inert atmosphere: (**A**) CRC1, (**B**) CRC2, (**C**) CRC3, (**D**) EAFSS1, (**E**) EAFSS2, (**F**) EAFSS3, (**G**) EAFCS1, (**H**) EAFCS2, and (**I**) EAFCS3.

The TG curves have significant mass reductions in three different temperature ranges in all three samples. The first range would be the dihydroxylation process and the partial decomposition of carbonate around the temperature range of 180 to 520 °C. The mass loss of each dust sample was increased with the increments of lignin proportion in this temperature range. This result is alongside the mass spectrum curves, particularly in the fluctuations of m18 and m44 across all dust samples (Appendix A: Figure A1) within this temperature range. These fluctuations signify the evaporation of water vapors and CO_2_, likely originating from dihydroxylation and partial decomposition processes. Moreover, CRC dust samples exhibit a minimum mass loss ranging from 6.10% to 10.43%, while EAFCS samples display the highest mass loss, varying from 9.49% to 20.77% in this temperature region, indicating that EAFCS dust contains more hydroxides and MgCO_3_ inside the sample. The next mass reduction process occurred from 464 to 771 °C. During this temperature range, just two vapors are identified (Appendix A: Figure A1): CO and CO_2_. These two gases are related to the decomposition of (CO_3_)^2−^, probably CaCO_3_. In the CRC and EAFCS dust samples, there is an almost positive correlation observed with the proportion of lignin and the mass loss, suggesting that the addition of volatile matters from lignin significantly contributes to the mass loss within this temperature range. Conversely, in EAFSS samples, a negative relationship is evident between the proportion of lignin and mass loss in this temperature range, which underscores the need for further research. The most important feature in this temperature region would be the comparatively higher mass loss in EAFCS samples, indicating a higher presence of carbonates and volatile matters within the EAFCS–lignin mixtures. The final mass reduction process was between 686 and 1200 °C for all three dust samples. CRC dust samples show escalating mass loss in this temperature region with the increments of lignin, resulting in the highest mass loss in CRC3. This suggests an ongoing reduction process, primarily due to iron reduction. In both EAFSS and EAFCS samples, the mass loss was decreased with the increments of lignin, indicating that the minimum lignin proportion would be enough for the complete reduction process. It was noticeable that CO has very high ionic current fluctuation, indicating that a significant amount of CO has been emitted and transferred into the MS analyzer, mainly due to the reduction process (Appendix A: Figure A1). There was some fluctuation in CO_2_ curve within the temperature region which means CO has been further oxidized into CO_2_.

**Figure 6:**
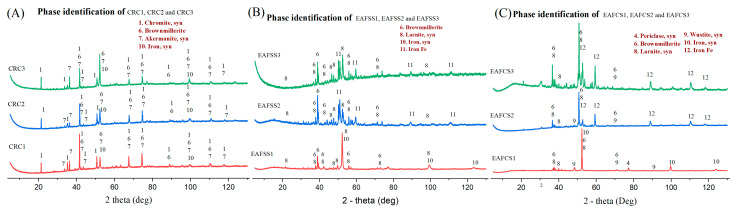
XRD analysis of CRC, EAFSS and EAFCS dusts with different lignin mixing ratios compared with raw steel dust in inert atmosphere: (**A**) CRC, (**B**) EAFSS and (**C**) EAFCS.

The XRD analysis of the residues of each steel dust sample was used to identify the remaining compositions inside each residue. As shown in Figure 6, the absence of any Zn-bearing mineral phases in all three types of dust–lignin mixtures suggests that all the zinc oxides underwent reduction into metallic Zn and subsequently evaporated due to the elevated temperature. In CRC dust samples, when the mixing proportion of lignin increases, the intensity of the metallic iron peak also increases. This finding suggests the requirement of additional lignin (fixed carbon) in the mixture for the complete reduction of iron oxide into metallic iron, which implies why the three CRC samples (CRC1, CRC2 and CRC3) had three different residue values after TG analysis, as shown Appendix A—Figure A2. In EAFSS dust samples, all three mixing ratios contain noticeable metallic iron peaks, with EAFSS1 exhibiting particularly prominent ones. This implies more lignin mixture may contaminate the sample with other mineral phases. In EAFCS samples, the presence of wustite is evident in all three samples, with a gradual decrease observed as the lignin proportion increases.

LECO test has been undergone to measure the remining carbon inside the samples after TG analysis and shown in Table 7 which gives a complete idea whether the fixed carbon has been enough for the complete reduction of Zn.

The CRC dust samples (CRC1, CRC2, and CRC3) exhibit the lowest C% within the residue post-TG analysis, indicating that the added fixed carbon (lignin) was insufficient for the complete reduction process within the specified temperature range. This observation is supported by the XRD results of CRC dust, shown in Figure 6A, which indicate gradually increasing peaks of metallic iron. However, the absence of zinc-bearing oxides in CRC indicates that even though the added fixed carbon was inadequate for complete iron reduction, it was sufficient for complete zinc reduction. Additionally, EAFSS and EAFCS samples exhibit a similar trend in C% within their residues, indicating that the C% increases with the mixing rate of lignin. Excessive C% is evident in both EAFSS and EAFCS samples under 1.3 and 1.5 stoichiometric ratios, suggesting that 1.1 would be the optimal lignin percentage among these three mixing ratios.

Following the identification of the minimum mixing ratio (1:1) as sufficient for the complete reduction process of zinc-bearing oxides into metallic zinc, the subsequent step involved analyzing the atmospheric impact on the reduction process of 1.1 lignin mixing samples.

### 3.4. Atmospheric Impact on Steel Dust–Lignin Mixture

**Figure 7:**
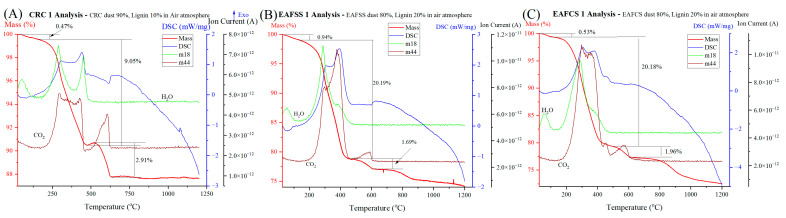
TG/DSC/MS curves of steel dust samples under air atmosphere: (**A**) CRC1, (**B**) EAFSS1, and (**C**) EAFCS1.

The influence of atmosphere was investigated using both air and inert (N_2_) atmospheres. The stages of mass losses, along with probable reactions and DSC behaviors, are outlined in Table 8. Furthermore, the graphical representation of the TG, DSC, and MS behaviors of three distinct steel dust samples (CRC1, EAFSS1, and EAFCS1) under an air atmosphere is highlighted in Figure 7.

Based on the TG/DSC/MS curves seen in Figure 7, the mass reduction zones under an air atmosphere can be divided into two main stages. The highest mass reduction occurred during the temperature range for all three dust samples, at around 100–500 °C. There were high fluctuations in the H_2_O curve (m18) and CO_2_ curve (m44). During this temperature region, the DSC curves show significantly higher exothermic peaks. The most possible reaction would be the combustion of hydrocarbons, which might appear in lignin with the presence of adequate oxygen in an air atmosphere while emitting water vapors and CO_2_. In this temperature range, CRC1 dust samples exhibited the least mass reduction, whereas EAFSS1 and EAFCS1 showed nearly identical mass reductions (Table 8). This can be attributed to the varying quantities of lignin materials utilized in the experiment according to their stoichiometric ratios. Specifically, CRC1 contained the lowest lignin mixture, amounting to 10 g, while EAFSS and EAFCS dusts each contained 20 g. In the 468–632 °C temperature region, a significantly lower mass reduction could be observed in all three dust samples accompanied by a distinct endothermic peak in the DSC curve. The mass spectrometry data indicated a significant fluctuation in the m44 curve, which corresponds to the presence of CO_2_ gas. Most probably this could be due to the decomposition of calcium carbonates, resulting in CO_2_ emission (Figure 7).

**Figure 8:**
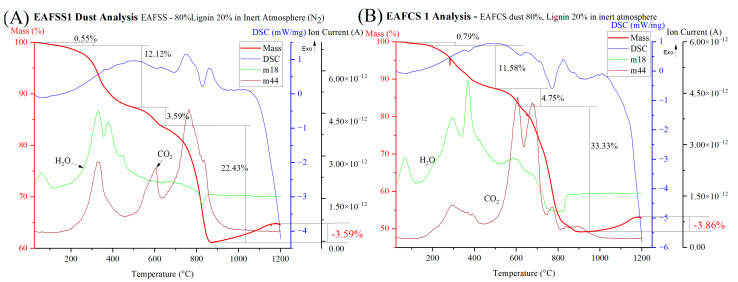
TG/DSC/MS curves of EAFSS1 and EAFCS1 under inert atmosphere: (**A**) EAFSS1 and (**B**) EAFCS1.

Moreover, in the TG, DSC, and MS curves of EAFSS1 and EAFCS1 under an inert atmosphere (as shown in Figure 8), distinct endothermic peaks are observable during the dihydroxylation and decomposition of the carbonate phases, occurring within temperature ranges of 200–500 °C and 500–600 °C, respectively.

Additionally, during the reduction stage, a combination of endothermic and exothermic peaks is present, likely attributed to simultaneous reduction and oxidation processes within this temperature range. The main difference between the air and inert atmospheres is evident in the MS curves, where under an air atmosphere, a plateau shape was observed after 700 °C, suggesting a minimal or no reduction process. In contrast, a significant mass loss occurs in this temperature region under an inert atmosphere, indicating complete reduction processes taking place.

**Figure 9:**
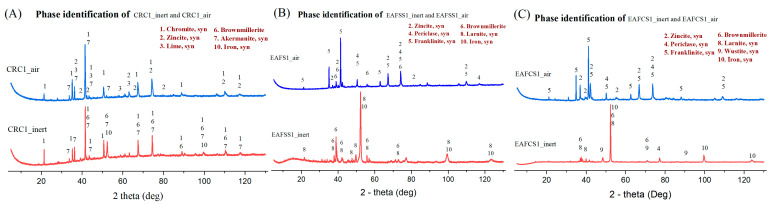
XRD results of CRC1, EAFSS1 and EAFCS1 under air and inert atmospheres compared with raw steel dusts: (**A**) CRC and (**B**) EAFSS, and (**C**) EAFCS.

The XRD results provide a clear indication of the reduction process in the steel dust samples under two different atmospheres. As shown in Figure 9, CRC1 exhibits prominent zincite peaks, while EAFSS1 and EAFCS1 show both zincite and franklinite phases under an air atmosphere, suggesting that the mixed lignin was not utilized for the zinc reduction process due to the combustion of the fixed carbon in the presence of oxygen. Conversely, in the inert atmosphere, the absence of zincite and franklinite phases implies that the fixed carbon in lignin was utilized for the reduction process of zinc oxides into metallic zinc and the evaporation of the metallic zinc due to the elevated temperature.

### 3.5. Effect of Lignin as a Reducing Agent Compared to Graphite

Once it was established that lignin mixing samples with a ratio of 1.1 under an inert atmosphere achieved complete zinc reduction, the subsequent step involved comparing this lignin mixture’s reduction capability with a reference graphite sample.

**Figure A3:**
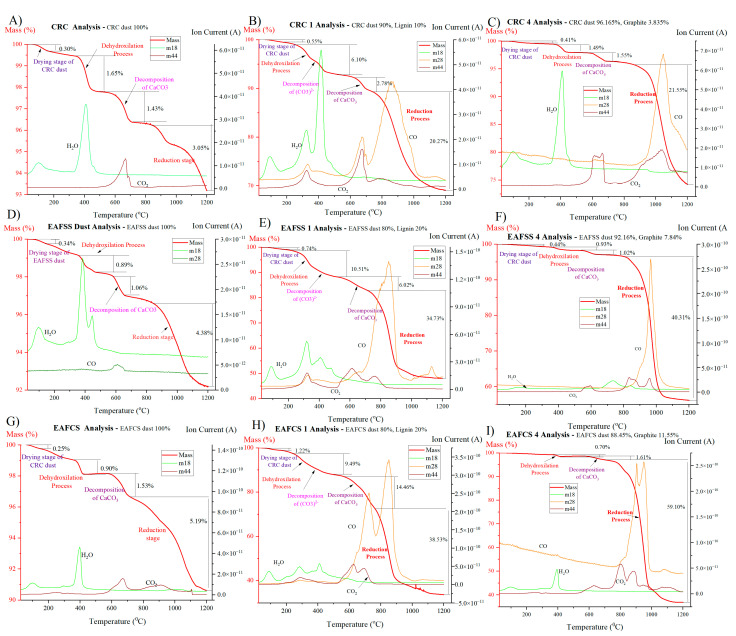
TG-MS analysis of different reducing agents with different dust samples under inert atmosphere: (**A**) CRC, (**B**) CRC1, (**C**) CRC4, (**D**) EAFSS, (**E**) EAFSS1, (**F**) EAFSS4, (**G**) EAFCS, (**H**) EAFCS1, and (**I**) EAFCS4.

The mass losses across three temperature ranges concerning different reaction processes with distinct reducing agents under an inert atmosphere are presented in Table 9. Additionally, the graphical representation of the TG and MS curves for each dust sample is provided in Appendix B: Figure A3. An identical observation was that the highest mass reduction was achieved in the lignin mixture for all three types of steel dust (CRC1, EAFSS1 and EAFCS1), which is higher than that of the graphite mixture. This is attributed to the higher volatile matter concentration in lignin. The mass reduction percentages for CRC1, EAFSS1, and EAFCS1 were 30.93%, 51.98%, and 66.35%, respectively, while those for graphite mixtures were 25.82%, 43.84%, and 63.09%, respectively. The highest mass reduction was observed in EAFCS mixtures for both reducing agents, possibly due to the highest zinc content in the EAFCS sample.

**Figure 10:**
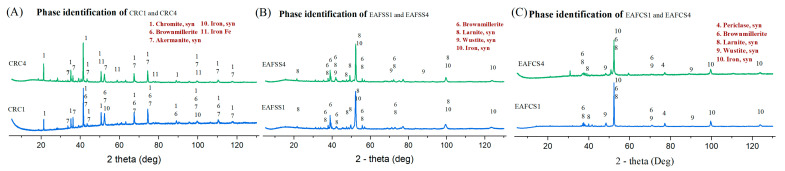
XRD results of each dust samples with different reducing agents: (**A**) CRC, (**B**) EAFSS, and (**C**) EAFCS.

The XRD pattern (Figure 10) indicated the absence of zinc-bearing phases in all three types of steel dusts in both lignin and graphite mixtures. This suggests that both the graphite and lignin mixtures were effectively utilized for the complete reduction process of zinc—the primary focus of this study. Thus, it was confirmed that the fixed carbon within the lignin was utilized similarly to the reference graphite sample for the zinc reduction process. Additionally, both reducing agents were employed for the reduction process of iron oxide, as evidenced by the identification of metallic iron peaks in both lignin and graphite samples in all three types of dust. This provides additional evidence that lignin can also be utilized for the reduction process of iron oxide and can serve the same function as the graphite mixture.

### 3.6. Morphological Observations of CRC1, EAFSS1 and EAFCS1 Dust under Inert Atmosphere

**Figure 11:**
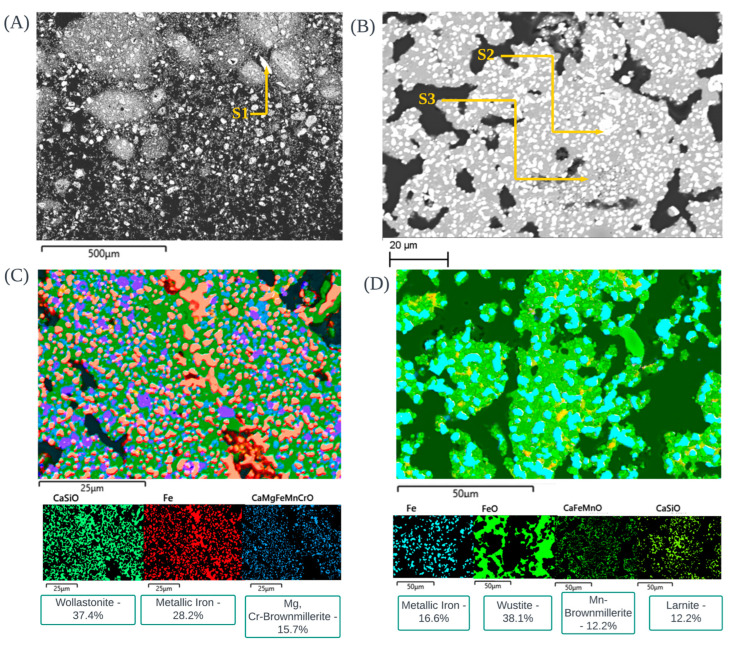
SEM images of CRC1 dust after TG (**A**), EAFSS1 dust after TG (**B**,**C**) and EAFCS1 dust after TG (**D**) under inert atmosphere: (i) Chromite phase covered with different fine-grained materials (Ca, Mg, Si, Cl) in CRC1 residue (S1), (ii) Metallic iron agglomerated with different alloys (Ni, Mn, Cr, Cu) (S2) and wollastonite phase (S3) in EAFSS1 residue, Possible phase distribution of EAFSS1 residue after TG analysis (**C**), (iii) Possible phase distribution of EAFCS1 residue after TG analysis (**D**).

The morphological observations of CRC1, EAFSS1 and EAFCS1 dust after TG analysis under an inert atmosphere are shown in Figure 11.

Irregular-shaped chromite phases exist in CRC1 residue, identified through Figure 11A (S1). Our major observation in these spectrums was the agglomeration of Mg, Ca, Si, and Cl fine-grained particles around the chromite phase. EDX spectrums do not show any Zn presence inside the CRC1 after TG sample.

The EAFSS1 after TG sample shows that the white color phases belong to metallic iron (in 19-B), which were enriched inside the sample—this implies the reduction process of franklinite into metallic iron (S2). This metallic iron was agglomerated with different fine-grained alloying particles such as Ni, Mn, Cr, and Cu. These metallic iron grains were enclosed by Mg-Mn-oxide, which should have originated from the wollastonite phase (S3). Manganese from the scrap and ferroalloys can oxidize to form MnO, which may react with available MgO to form mixed Mg-Mn oxides. This Mg-Mn-O phase is chemically stable at high temperatures, enabling it to encapsulate contaminants and impurities from the dust, thereby facilitating a well-directed reduction process of Zn oxide. Figure 11C shows the possible phase distribution of EAFSS1 residue after TG analysis, with no presence of Zn. The EAFCS1 after TG sample in Figure 11D shows the possible phase distribution of EAFCS1 residue after TG analysis which indicates the presence of metallic iron, wustite, brownmillerite and larnite phases with no clue of presence of Zn.

## 4. Conclusions

The study investigated three types of steel dust byproducts: CRC, EAFSS, and EAFCS. CRC dust exhibited high chromite content alongside zincite as a main phase, while both EAFSS and EAFCS dust contained significant franklinite and zincite phases. EAFSS dust showed elevated Ni and Mn content due to the input of stainless scraps, while EAFCS dust had the highest Zn content from carbon steel scraps. Lignin, with 28.9% fixed carbon, was chosen as the reducing agent and compared to graphite. TG/DSC/MS and XRD analysis revealed lignin’s similar reduction capacity to graphite when adequate fixed carbon was present. Lignin emerges as a promising alternative for reducing zincite and franklinite to metallic iron and zinc. The stoichiometric ratio of a 1:1 mixing proportion proved sufficient for complete zinc reduction in all dust types. The reduction reaction commences around 750 °C, accompanied by a significant mass reduction observed until 1000 °C. Residual materials showed intense metallic iron peaks, indicating lignin’s capability to partially reduce iron oxide. Due to lignin’s combustible nature, an inert atmosphere is crucial for its effective utilization as a reducing agent.

## Figures and Tables

**Table 1:** Stoichiometric mixing ratios of dust, lignin, and graphite for experiments.

Type	Sample	Reducing Agent	Stoichiometric Amount
CRC	CRC	-	-
	CRC1	lignin	1.1
	CRC2	lignin	1.3
	CRC3	lignin	1.5
	CRC4	graphite	1.3
EAFSS	EAFSS	-	-
	EAFSS1	lignin	1.1
	EAFSS2	lignin	1.3
	EAFSS3	lignin	1.5
	EAFSS4	graphite	1.3
EAFCS	EAFCS	-	-
	EAFCS1	lignin	1.1
	EAFCS2	lignin	1.3
	EAFCS3	lignin	1.5
	EAFCS4	graphite	1.3

**Table 2:** Chemical composition of CRC, EAFSS and EAFCS dust samples.

	CRC	EAFSS	EAFCS
Element	Major Elements (wt.%)		
C	0.3	0.5	1.5
Zn	10.83	19.84	35.76
Cr	20.88	3.19	0.47
Fe	18.74	23.70	23.50
MgO	9.76	7.21	1.07
MnO	1.56	5.82	3.99
CaO	14.27	11.91	5.93
Al_2_O_3_	0.80	0.87	0.27
K_2_O	0.74	1.49	3.21
P_2_O_5_	0.02	0.08	0.13
Cl	0.29	1.25	1.69
F	0.31	0.51	0.37
TiO_2_	0.09	0.09	0.05
Pb	0.11	0.62	2.15
Ni	0.25	0.37	0.038
S	0.14	0.48	1.22
Cu	0.032	0.30	0.25
pH	10–11	11	10–11
Moisture %	0.4	0.8	0.98

**Table 3:** Ultimate and proximate analysis of lignin [31].

Properties of Hydrolysis Lignin				Standard/Analysis Methods
Total moisture (105 °C)		[m%]	5.3	SFS-EN 14774-2 [32], CEN/TS 15414-2 [33], ISO 589 [34]
Ultimate analysis	C	%, d. b.	61	SFS-EN ISO 16948 [35], SFS-EN 15407 [36], ISO 29541 [37]
	H	%, d. b.	6.1	SFS-EN ISO 16948 [35], SFS-EN 15407 [36], ISO 29541 [37]
	O	%, d. b.	31.9	SFS-EN ISO 16993 [38]
	N	%, d. b.	0.69	SFS-EN ISO 16948 [35], SFS-EN 15407 [36], ISO 29541 [37]
	S	%, d. b.	0.12	ASTM D 4239 (mod) [39], SFS-EN ISO 16994 [40]
Proximate analysis	Volatile matter	%, d. b.	70.9	SFS-EN ISO 18123 [41], SFS-EN 15402 [42], ISO 562 [43]
	Ash content	%, d. b.	0.2	SFS-EN ISO 18122 [44], SFS-EN 15403 [45], ISO 1171 [46]
	Fixed Carbon	%, d. b.	28.9	Determined by difference

**Table 4:** Chemical composition of ash content of lignin [31].

	Ash Content	
Element	unit	amount
Cl	%, d. b.	0.002
F	%, d. b.	<0.001
Br	%, d. b.	<0.001
Ca	mg/kg, d. b.	290
Mg	mg/kg, d. b.	51
Na	mg/kg, d. b.	<10.000
K	mg/kg, d. b.	130
P	mg/kg, d. b.	74
S	mg/kg, d. b.	1100
Fe	mg/kg, d. b.	810
Al	mg/kg, d. b.	14
Si	mg/kg, d. b.	<10.000
Ti	mg/kg, d. b.	<1.000
Mn	mg/kg, d. b.	41
Ba	mg/kg, d. b.	4.8
Cr	mg/kg, d. b.	15
Cu	mg/kg, d. b.	<1.000
Ni	mg/kg, d. b.	12
Zn	mg/kg, d. b.	12
Pb	mg/kg, d. b.	1.3
V	mg/kg, d. b.	<1.000
Ar	mg/kg, d. b.	<0.500

**Table 5:** Elemental compositions of selected spectrums highlighted in Figure 3.

Elements	wt%									
	S1	S2	S3	S4	S5	S6	S7	S8	S9	S10
	Mg-Mn-Si- Chromite	Zincite	Franklinite-Glass Sphere	Franklinite-Glass Sphere	Ca-Franklinite	Ca-Franklinite	Zincite	Zincite	Franklinite	Franklinite
O	31.29	21.40	32.19	30.51	32.06	29.07	20.73	22.37	24.83	26.64
Zn		33.82	11.58	19.2	8.29	14.46	58.36	62.45	19.38	19.87
Ca			21.21		16.22	9.25				
Fe	13.86	3.34	10.14	12.92	32.63	37.13			32.13	34.45
Mg	3.42			19.55						
Cr	40.54	6.23								
Mn	1.21									
Na					3.67	1.98				
C		28.79								

**Table 6:** Different stages of mass losses under inert (Ar) atmosphere.

Sample	Dihydroxylation and Partial Decomposition			Decomposition of Carbonates			Reduction			Total Mass Loss %
	Start °C	End °C	Mass Loss %	Start °C	End °C	Mass Loss %	Start °C	End °C	Mass Loss %	
CRC1	257	520	6.10	627	745	2.78	745	1200	20.27	30.93
CRC2	248	457	8.01	573	717	3.27	778	1200	22.48	37.31
CRC3	265	519	10.43	519	739	4.01	739	1200	26.70	43.04
EAFSS1	220	519	10.51	519	732	6.02	732	1200	34.73	51.98
EAFSS2	243	464	11.50	464	692	5.70	692	1200	34.82	54.21
EAFSS3	236	478	14.04	538	686	4.23	686	1200	32.79	54.21
EAFCS1	247	502	9.49	565	765	14.46	765	1200	38.53	66.62
EAFCS2	184	474	14.77	474	754	14.34	754	1200	37.73	68.54
EAFCS3	243	481	20.77	481	771	14.70	771	1200	29.71	66.62

**Table 7:** LECO analysis steel–lignin dust samples under inert atmosphere after TG analysis.

**Sample**	CRC1	CRC2	CRC3	EAFSS1	EAFSS2	EAFSS3	EAFCS1	EAFCS2	EAFCS3
C% (wt)	0.10	0.18	0.81	0.20	3.94	8.59	0.25	12.23	28.87

**Table 8:** Different stages of mass losses of 1.1 lignin mixing ratio under air atmosphere.

Sample	Combustion of Hydrocarbon				Decomposition of Carbonates			
	Start °C	End °C	Mass Loss %	DSC	Start °C	End °C	Mass Loss %	DSC
CRC1	155	477	9.05	exothermic	521	632	2.91	endothermic
EAFSS1	100	456	20.19	exothermic	456	610	1.69	endothermic
EAFCS1	97	468	20.18	exothermic	468	607	1.96	endothermic

**Table 9:** Different stages of mass losses under different reducing agents.

Sample	Reducing Agent	Dihydroxylation and Partial Decomposition			Decomposition of Carbonates			Reduction			Total Mass Loss %
		Start °C	End °C	Mass Loss %	Start °C	End °C	Mass Loss %	Start °C	End °C	Mass Loss %	
CRC	no	100	494	1.65	494	717	1.43	845	1200	3.05	6.82
CRC1	lignin	257	520	6.10	627	745	2.78	745	1200	20.27	30.93
CRC4	graphite	211	470	1.49	470	688	1.55	666	1200	21.55	25.82
EAFSS	no	357	474	0.89	583	658	1.06	843	1200	4.38	7.82
EAFSS1	lignin	220	519	10.51	519	732	6.02	732	1200	34.73	51.98
EAFSS4	graphite	355	530	0.93	530	320	1.02	806	1200	40.31	43.84
EAFCS	no	366	433	0.90	562	719	1.53	719	1200	5.19	9.40
EAFCS1	lignin	247	502	9.49	565	765	14.46	765	1200	38.53	66.62
EAFCS4	graphite	367	413	0.70	538	698	1.61	698	1200	59.10	63.09

**Figure A2:**
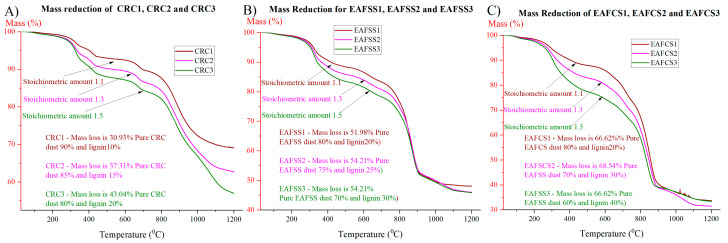
Mass loss comparison of CRC, EAFSS and EAFCS with different lignin mixing ratios under inert atmosphere: (**A**) CRC, (**B**), EAFSS and (**C**) EAFCS dust samples.

## Data Availability

The original contributions presented in the study are included in the article, further inquiries can be directed to the corresponding authors.
